# The Nervous System

**Published:** 1893-11

**Authors:** Leander J. White


### THE NERVOUS SYSTEM.

There is no subject perhaps which is so often mentioned, but so little understood by the public in general, as that of the "nerves." How often do we hear all classes of the community refer any unpleasant sensation or fanciful ailment to their being merely nervous ; little understanding, however, when they make use of this term, what possible connection there can be between their feelings and their *nervous* system.

Perhaps we shall surprise them when we mention that they can neither eat or drink, walk or talk, nor perform any action whatever, either voluntary or involuntary, but through the medium of their nervous system—a "system" the nature and functions of which we shall here endeavor to explain.

In man and other vertebrate animals, the great centre of the function is the brain and spinal marrow ; the latter a prolongation of the brain, as it were, down the spine.

Now this great centre of nervous matter is endowed with two distinct functions. First—that of being able to convey *motor power* to the muscles, by whose agency we are enabled to perform all the ordinary actions of the body, all the movements of our limbs. Second, that of sensation, which is of two kinds—*common sensation*, or that feeling of pain which is produced on the injury of any part of our body ; and *special sensation*, to which are to be referred the five senses of feeling, of hearing, of smelling, and of taste. From this mass of matter, capable of endowing the parts of our bodies with the power of motion, and of feeling or sensation, numerous trunks are sent off to all parts of the human frame—ramifying over its structure to such an inconceivable state of minuteness, that we cannot touch any part of our body with even the point of a needle without being conscious of pain, proving that some part of this great nervous centre has been injured or excited into action.

The great nervous trunk which supplies the lower extremity of man is equal in thickness to his little finger; divide it, and he loses all power of moving his limb, all sense of feeling ; the limb to all intents and purposes is dead ; and, deprived of its nervous influence, mortifies.

This power of endowing parts with motion and sensation is situated in two distinct structures, of which the brain and spinal marrow are composed ; and anatomists, from their color, are accustomed to call them the *white* and the *gray* matter.

In the brain, the gray matter for the most part is external, enclosing in its folds the white matter ; whilst in the spinal marrow it is internal, being completely surrounded by the white. Now, as a general rule, all the nervous trunks of the body and their branches, with the exception of nerves of special sensation, are composed of fibres derived from these two sources—that is from the *white* and *gray* matter ; and these nervous trunks are conductors of that *change* produced in the nervous centre by the influence of the *mind*, which gives rise either to motion or sensation.

But a most extraordinary fact, and one which is capable of being proved by direct experiment, is, that the change which takes place, to give rise to the phenomena of motion, has its origin at the great nervous centre, the source from which the trunks arise ; and further, that this change takes place in the white matter. On the other hand, the change which gives rise to the phenomena of sensation takes place at the extremities of the nervous trunks—that is, at their ultimate distribution ; and this change takes place in the gray matter.

The anatomist, in his dissections, is able to prove satisfactorily the origin of these nervous trunks ; and he finds that all those arising from the spinal marrow, and most of those which are said to arise from the brain, do so by two roots, one of which is connected with the white matter, and the other with the gray. He can, and has still further proved by experiments performed on the living animals, that irritation by pinching or pricking of the root which arises from the white matter gives rise to no sensation, as the animal shows no signs of suffering whatever ; but irritate the root arising from the gray matter, and evident signs of suffering are immediately induced.

Again : if in the dead animal we excite muscular contraction by means of galvanism, we must send the charge of electricity through the limb by means of the root arising from white matter, as no effect would be produced if we attempted to do it by means of the root arising from the gray matter. Allowing, then, the fact, that these nervous trunks are composed of two sets of fibres, one conveying sensitive, the other motor influence, let us apply it to practice.

Some part of the body meets with an injury—a change is immediately effected in the extremities of the sentient fibres, sensation is developed, and the change is developed, and the change thus induced is conveyed by the sentient fibre of the brain, and through its medium of the mind.

Through the mysterious agency of the mind, then, the motor power of the great nervous centre is brought into action, and a change is induced ; this change is conveyed by the trunks to the muscles supplying the injured parts, or to other muscles by whose combined action it is removed from further injury. But it is not necessary that an injury should be inflicted that motor influences should be generated, as the mind has the power of inducing it at will.

All the movements of our bodies are effected by muscular action, and through the agency of the will.

We move not a hand or foot, nor look at an object, without the mind having first willed that it shall be done. But there are many actions in the human body which are performed independently of the will, though evidently under the influence of the mind, and through the medium of a nervous system; and this system is called by the anatomist the *sympathetic*. It consists of a number of little knot-like bodies, called by the anatomist *ganglia*, which are extended along each side of the vertebral column, the whole of these ganglia being connected, by means of fibres, together. Now it appears that each of these ganglia is capable of generating nervous influence, independently of the brain; hence each may be considered as a distinct nervous centre.

The trunks arising from these ganglia are distributed principally to all those organs on which the vitality of the body depends, which are employed in secretion and its nutrition.

It is the medium by which all parts of the body are brought into relation with each other, so that no one part shall become diseased or injured without the rest sympathizing with it, and indirectly, therefore, becoming effected as well. Familiar examples of this fact are of every day occurrence. A violent blow on the head will produce vomiting.

Owing to the sympathy which exists between the brain and the stomach, and *vice versa*, a blow on the stomach will produce fainting, and even death, from the shock to the nervous system, and the arrest of its influence through the medium of the brain.

And now let us turn our attention once more to the influence of the mind over the functions of the body, through the agency of this part (the sympathetic) of the nervous system. We will here select a few familiar examples. What is referred to when one's mouth is said to be "watery" at the sight of some favorite fruit or food, is dependent on the influence of the mind acting through the medium of the nervous system applying the organs secreting the saliva. Tears, again, are abundantly secreted under the moderate exciting influence of the emotions of joy, grief, or tenderness.

When, however, the exciting cause is violent, they are suppressed; hence in excessive grief, the anguish of the mind is lessened on the flow of tears. Fear stops the flow of saliva; and it is a common practice in India to detect a thief among the native servants by putting rice into their mouths, and he whose mouth is driest after a short time is considered the culprit. Under mental anxiety, persons become thin; freedom from it favors deposit of fat. It would be an endless task, however, to recapitulate the many examples that could be brought forward proving this influence of the mind; so that nervous complaints must be looked upon as disorders of the mind, and not of the body; cure the one and you will cure the other.

*Mental influence* having then this power over the functions of the body, we cannot be surprised at many diseases being a consequence of its depraved or abnormal condition. Nor can we be surprised at many of the remarkable phenomena displayed by mesmerists; their patients, on whom they exhibit, are generally highly sensitive, with minds naturally liable to become excited under the manipulations of the operator.

For this reason also, homeopathy, hydropathy, etc., have succeeded in curing many patients of their fancied ailments, because it only required some strong excitement to remove the morbid mental impression. Hence change of scene and diet, change of usual habits (for all the followers of these systems make it imperative on their patients to follow implicitly certain rules), and lastly, and not *least*, a full determination, desire, or will on the part of the patient himself to get better—have succeeded in a variety of complaints arising from mental causes, in effecting a cure. LEANDER J. WHITE.

